# Cyber-resilience drivers in digital business networks: an expert-based prioritization using dual-gray DEMATEL and adaptive SWARA

**DOI:** 10.3389/frai.2026.1863919

**Published:** 2026-08-05

**Authors:** Reem Aljuaidi, Mohammed Alkhowaiter, Mohamed Ayari, Majed S. Alsayfi, Eman Abouelkheir, Wedad M. Alawad, Mohammad Ahmed Alnakhli

**Affiliations:** 1 Prince Sattam Bin Abdulaziz University College of Computer Science and Engineering, Al-Kharj, Saudi Arabia; 2 Northern Border University, Arar, Saudi Arabia; 3 Taibah University College of Engineering, Madinah, Saudi Arabia; 4 Qassim University, Buraydah, Saudi Arabia

**Keywords:** cyber resilience, decision support, digital business networks, Dual-Gray DEMATEL, SWARA

## Abstract

**Introduction:**

Digital business networks are increasingly exposed to complex cyber threats, making the identification and prioritization of cyber resilience drivers essential for organizational stability. This study aims to identify, structure, and prioritize key cyber resilience drivers relevant to digital business network environments.

**Methods:**

To achieve this objective, an expert-based multi-criteria decision-making framework was developed by integrating Dual-Grey DEMATEL and Adaptive SWARA. Dual-Grey DEMATEL was used to analyze the interrelationships among resilience drivers under uncertainty, while Adaptive SWARA was applied to determine their relative importance based on expert evaluations.

**Results:**

The findings reveal the most influential drivers that organizations should prioritize to strengthen cyber resilience capabilities within interconnected digital ecosystems.

**Discussion:**

The study provides a structured decision-support perspective for managers and researchers seeking to improve cyber resilience planning in digitally connected business environments. Methodologically, this exploratory study contributes by integrating grey-based causal analysis with adaptive weighting to support resilience-oriented decision-making under uncertainty.

## 1 Introduction

Digital transformation has fundamentally reshaped how organizations operate, interact, and create value within modern business ecosystems (Tsai et al., 2022; Verhoef et al., 2021). The increasing adoption of interconnected platforms, cloud infrastructures, and data-driven technologies including AI-enabled systems has improved organizational efficiency, innovation capability, and operational flexibility (Vial, 2019; Tsai et al., 2022). However, growing digital interconnectedness has also increased exposure to cyber threats capable of disrupting operations, compromising sensitive information, and affecting interconnected business environments (Saeed et al., 2023; Chandna and Tiwari, 2023; Kure et al., 2022). As cyber incidents become more sophisticated and difficult to prevent entirely, organizations increasingly require capabilities that enable them not only to resist disruptions but also to maintain continuity and recover effectively from adverse events (Bagheri et al., 2023; Kott and Linkov, 2019).

In this context, cyber resilience has emerged as a critical organizational capability (Linkov et al., 2013; Barasa et al., 2018). Cyber resilience refers to the ability of organizations to anticipate, withstand, respond to, and recover from cyber disruptions while sustaining essential operational functions (Barasa et al., 2018; Kott and Linkov, 2019). Unlike traditional cybersecurity approaches that primarily emphasize prevention, cyber resilience focuses on adaptability, continuity, recovery, and organizational learning during and after disruptive events (Annarelli et al., 2020; Bhamra et al., 2011). This perspective reflects the recognition that complete protection against cyber threats is unrealistic in highly interconnected digital systems, particularly in AI-enabled ecosystems where threats can propagate rapidly (Hausken, 2020; Kott and Linkov, 2021). Consequently, organizations must strengthen technological, organizational, strategic, and collaborative capabilities that support rapid response, coordinated decision-making, and operational recovery following cyber incidents (Urciuoli, 2015; Annarelli and Nonino, 2016).

Existing studies suggest that cyber resilience depends on multiple interrelated capabilities operating at technological, organizational, and network levels (Annarelli and Palombi, 2021; Linkov et al., 2019). Technological capabilities such as monitoring systems, infrastructure maturity, and security mechanisms including AI-driven threat detection and intelligence integration improve the detection and containment of cyber incidents (Saeed et al., 2023; Zaman et al., 2023). Organizational capabilities including crisis leadership, adaptive decision-making, and knowledge sharing enhance coordination and flexibility during disruptions (Duchek, 2020; Gündüzyeli, 2025). In addition, network-level capabilities such as collaboration, situational awareness, and information exchange contribute to resilience across interconnected ecosystems and digital supply chains (Tagarev and Yanakiev, 2020; Wieland and Durach, 2021). Because digital business networks are highly interconnected, weaknesses in one component may rapidly propagate across partner organizations and supply chains, generating broader operational and financial consequences (Dolgui et al., 2020; Sukachova et al., 2025).

Despite increasing scholarly attention to cyber resilience, important limitations remain in the existing literature (Bagheri et al., 2023; Annarelli et al., 2020). Many studies focus primarily on conceptual discussions without systematically examining the interrelationships among resilience drivers within complex digital environments, particularly AI-enabled digital business ecosystems (Kott and Linkov, 2019). Moreover, although prior research has identified numerous resilience-related factors, relatively few studies prioritize these drivers according to their strategic importance while simultaneously accounting for uncertainty in expert evaluations (Moşteanu, 2020; Korucuk et al., 2022). Existing research also tends to examine technological, organizational, and collaborative dimensions separately, limiting the development of integrated analytical perspectives suitable for digitally interconnected ecosystems (Chandna and Tiwari, 2023; Gudla and Jamalpur, 2024). Consequently, organizations still lack structured decision-support frameworks capable of analyzing both the causal relationships and relative importance of cyber-resilience drivers under uncertain conditions.

To address these limitations, this study integrates Dual-Gray DEMATEL and Adaptive SWARA to prioritize cyber-resilience drivers in digital business networks, with particular attention to AI-enabled ecosystems. Dual-Gray DEMATEL is employed to model the causal relationships among resilience drivers while preserving uncertainty and ambiguity in expert judgments through gray interval numbers. The resulting influence structure is subsequently incorporated into the Adaptive SWARA procedure to determine the relative importance of the identified drivers. Unlike conventional DEMATEL-SWARA approaches that rely on crisp evaluations, the proposed framework provides a more uncertainty-aware and structurally informed prioritization process.

This study makes three distinct contributions to the literature on cyber resilience in digital business networks. Conceptually, it advances a systemic view of cyber resilience drivers in AI-enabled digital business networks, framing resilience not merely as protection against discrete incidents but as an evolving organizational capability that spans technological, organizational, and human-cultural domains a perspective particularly relevant for ecosystems where AI plays a central role in monitoring, threat intelligence, and adaptive response. Methodologically, it introduces an integrated framework that combines Dual-Gray DEMATEL prominence analysis with adaptive SWARA weighting under conditions of uncertainty, providing a more nuanced prioritization mechanism than standalone MCDM methods by incorporating expert-perceived influence structures directly into the weighting procedure. Practically, it delivers a prioritization roadmap for organizations operating in AI-enabled digital ecosystems, highlighting which resilience drivers require strategic attention based on both their influence within the network and their relative importance, thereby supporting evidence-based resource allocation and capability development in complex, interconnected environments. It is important to clarify that while AI capabilities (such as threat intelligence integration and AI-driven monitoring) are examined as key resilience drivers, the methodological approach itself relies on structured expert judgment rather than AI algorithms, positioning this work within the “AI in Business” domain through its focus on resilience in AI-enabled ecosystems rather than through AI methodological contributions.

It is important to clarify the positioning of this study within the “AI in Business” domain: while artificial intelligence (AI) appears as critical components within several resilience criteria (notably C10 “Threat intelligence integration” and C13 “AI-driven monitoring”), the methodological contribution lies in the integration of expert-based Multi-Criteria Decision Making (MCDM) techniques specifically Dual Gray DEMATEL and adaptive SWARA rather than in the development or application of AI algorithms *per se*. The framework leverages structured expert judgment to prioritize capabilities that support resilience as an ongoing, systemic process in AI-enabled environments.

Accordingly, this study aims to identify and prioritize the key drivers influencing cyber resilience in digital business environments. Based on a systematic literature review and expert validation, 17 resilience criteria were identified and analyzed using the proposed framework. The findings highlight the drivers that appear most influential within digital business networks and therefore may require greater managerial attention and strategic investment. In doing so, the study contributes to the cyber-resilience literature and provides practical decision-support insights for organizations operating in digitally interconnected environments. Accordingly, this study addresses the following research questions:

*RQ1*. What are the key drivers influencing cyber resilience in digital business networks?
*RQ2*. How do cyber-resilience drivers influence one another within interconnected digital business environments?
*RQ3*. Which cyber-resilience drivers are the most critical when evaluated through an uncertainty-aware prioritization framework?

## 2 Literature review

### 2.1 Cyber-resilience drivers and theoretical framing

According to the dynamic capabilities framework (Barasa et al., 2018; Annarelli et al., 2020), companies can maintain a competitive advantage by skillfully coordinating special resources and talents that are unusual, valuable, hard to replicate, and non-replaceable (Karimi, 2010). The foundation of this viewpoint is competencies, which are seen as integrated sets of knowledge and abilities that allow organizations to coordinate activities and take advantage of resources (Annarelli and Palombi, 2021). Cyber-resilience drivers, which include the ability to maintain operational integrity amid cyber disruptions, are essential for differentiation and endurance in threat-laden environments within digital business networks interconnected setups like supply chains, cloud infrastructures, and collaborative platforms (Hausken, 2020; Urciuoli, 2015). Scholarship on cyber-resilience drivers in networked environments is still fragmented, with little focus on their structural delineation and reciprocal implications, despite a wealth of research on organizational capabilities. This section identifies an initial set of 13 drivers drawn from existing literature. After expert consultation (documented in “Research Gaps and Justification for Criteria Selection”), four additional human and cultural capabilities (C14–C17) were added. Consequently, the final analysis is based on 17 cyber-resilience drivers, which are grouped into four main categories as presented in Tables 1, 2.

**Table 1:** Expert panel composition.

Expert code	Domain of expertise	Years of relevant experience	Role/affiliation
E1	Cybersecurity strategy, digital risk management	18	Senior executive, financial services
E2	IT governance, compliance (ISO 27001)	22	IT director, manufacturing
E3	Threat intelligence, SOC operations	16	Lead analyst, security provider
E4	Cloud security, network infrastructure	19	Cloud architect, telecom
E5	Business continuity, crisis management	21	BCM manager, logistics
E6	Human factors in cybersecurity, training	17	HR director, retail
E7	Supply chain cyber resilience	20	Procurement head, automotive
E8	AI/ML for security monitoring	15	Data scientist, tech firm
E9	Regulatory compliance, data protection	24	Legal/compliance officer, healthcare
E10	Digital business ecosystems, platform governance	20	Academic researcher (professor)

To assess inter-rater agreement, Fleiss' kappa was calculated on the categorical ratings (before gray conversion), yielding K = 0.73, indicating substantial agreement. This supports the reliability of the aggregated judgments.

**Table 2:** Linguistic evaluation scale used for expert judgments.

Linguistic term	Gray interval
No influence	[0, 0]
Very low	[0, 1]
Low	[1, 2]
Medium	[2, 3]
High	[3, 4]
Very high	[4, 5]

### 2.2 Evolution of cyber-resilience research

The study of cyber-resilience has evolved from an early emphasis on physical and material resources toward a more nuanced understanding of intangible and adaptive capabilities (Sharma et al., 2024). Within the dynamic capabilities' tradition, organizational performance is explained not only by the possession of resources but also by the ability to sense threats, seize opportunities, and transform routines in response to changing conditions (Loonam et al., 2020; Sadeghi et al., 2024). As cyber threats have become more frequent and sophisticated, research has increasingly focused on resilience-specific capabilities that support continuity, adaptation, and recovery in digitally mediated environments (Gudla and Jamalpur, 2024). Recent studies further suggest that resilience should be examined as a multi-dimensional construct because cyber-resilience drivers often operate across operational, technological, human, and governance domains simultaneously, generating value for both organizations and their stakeholders (Sukachova et al., 2025; Kanaan et al., 2025). However, much of the existing research still examines resilience in isolation or at the level of large firms, while comparatively little attention has been paid to the interdependencies among drivers in networked settings (Zaman et al., 2023; Saeed et al., 2023). This limitation creates a need for an integrated review that clarifies the structural composition of cyber-resilience and the relative importance of its underlying criteria.

### 2.3 Dimensions and criteria of cyber-resilience drivers

Four categories may be used to classify cyber-resilience drivers: organizational and process, technology and infrastructural, human and cultural, and leadership and governance (Gündüzyeli, 2025; Tagarev and Yanakiev, 2020). Each category contains certain signs that, when taken as a whole, enable networks to provide strong defenses. The study concentrated on 17 criteria that were taken from previous research and honed in a thorough 3-h session with 10 experts. Among these 17 criteria, 13 were derived directly from the literature review (C1–C13), while the remaining four (C14–C17: awareness training programs, cultural embedding, behavioral risk mitigation, and team collaboration enhancement) were added based on expert recommendations to ensure full coverage of human and cultural dimensions.

### 2.4 Leadership and governance capabilities

According to Loonam et al. (2020), these are related to the senior-level directives, policies, and supervision that shape strategic responses and guide network performance. The literature identifies critical indications that are essential for digital networks, where danger posture is determined by oversight:

- Strategic risk assessment: to align defenses with network demands, Chandna and Tiwari (2023) stress executive competence in measuring and assessing cyber risks.
- Adaptive policy formulation: according to Moşteanu (2020), innovation in governance protocols maintains edges.
- Compliance integration: decision robustness is improved by proficiency in integrating regulatory adherence (Sukachova et al., 2025).
- Stakeholder alignment: leaders create networks that facilitate resource sharing and collaborative activities (Tagarev and Yanakiev, 2020).
- According to Kanaan et al. (2025), crisis leadership include directive styles that motivate and coordinate reactions during disturbances.

### 2.5 Organizational and process capabilities

These show how well an organization can combine and manage resources and procedures to achieve operational resilience (Zaman et al., 2023). The body of research recognizes the following as crucial for network defense scaling:

- Business continuity planning: Saeed et al. (2023) emphasize procedures for continuous operations in the face of disruptions.
- Incident response orchestration: according to Annarelli et al. (2020), streamlined procedures increase the effectiveness of recovery.
- Vendor risk management: controlling exposures to third parties increases defensive flexibility (Bagheri et al., 2023).
- Adaptive process redesign: according to Urciuoli (2015), decentralized configurations increase threat management agility.

### 2.6 Technological and infrastructure capabilities

These include the systems and technologies that convert resources into useful information that reduces threats (Annarelli and Palombi, 2021). Indicators that are essential for network acquisition and retention include:

- Integration of threat intelligence: Gündüzyeli (2025) emphasizes analytics to anticipate weaknesses.
- Network segmentation: according to Hausken (2020), segregated designs prevent the spread of breaches.
- Cloud security management: access and integrity are guaranteed by efficient controls (Zhu et al., 2020).
- AI-driven monitoring: proactive precautions are fueled by automated detection, as in (Sadeghi et al., 2024).

### 2.7 Human and cultural capabilities

These indicate how well the organization fosters defenses that are alert, responsive, and client-focused (Loonam et al., 2020). Indicators that are especially important in networks where loyalty is fueled by trust include:

- Programs for raising awareness: interactions that improve perceptions (Saeed et al., 2023).
- Cultural embedding: experiences are shaped by the surroundings (Loonam et al., 2020).
- Mitigation of behavioral risk: trust is shaped by the caliber of interpersonal interactions during defenses (Gudla and Jamalpur, 2024).
- Improving teamwork: credibility is reinforced by a positive image (Annarelli and Palombi, 2021).

## 3 Research methodology

### 3.1 Research design

**Figure 1:**
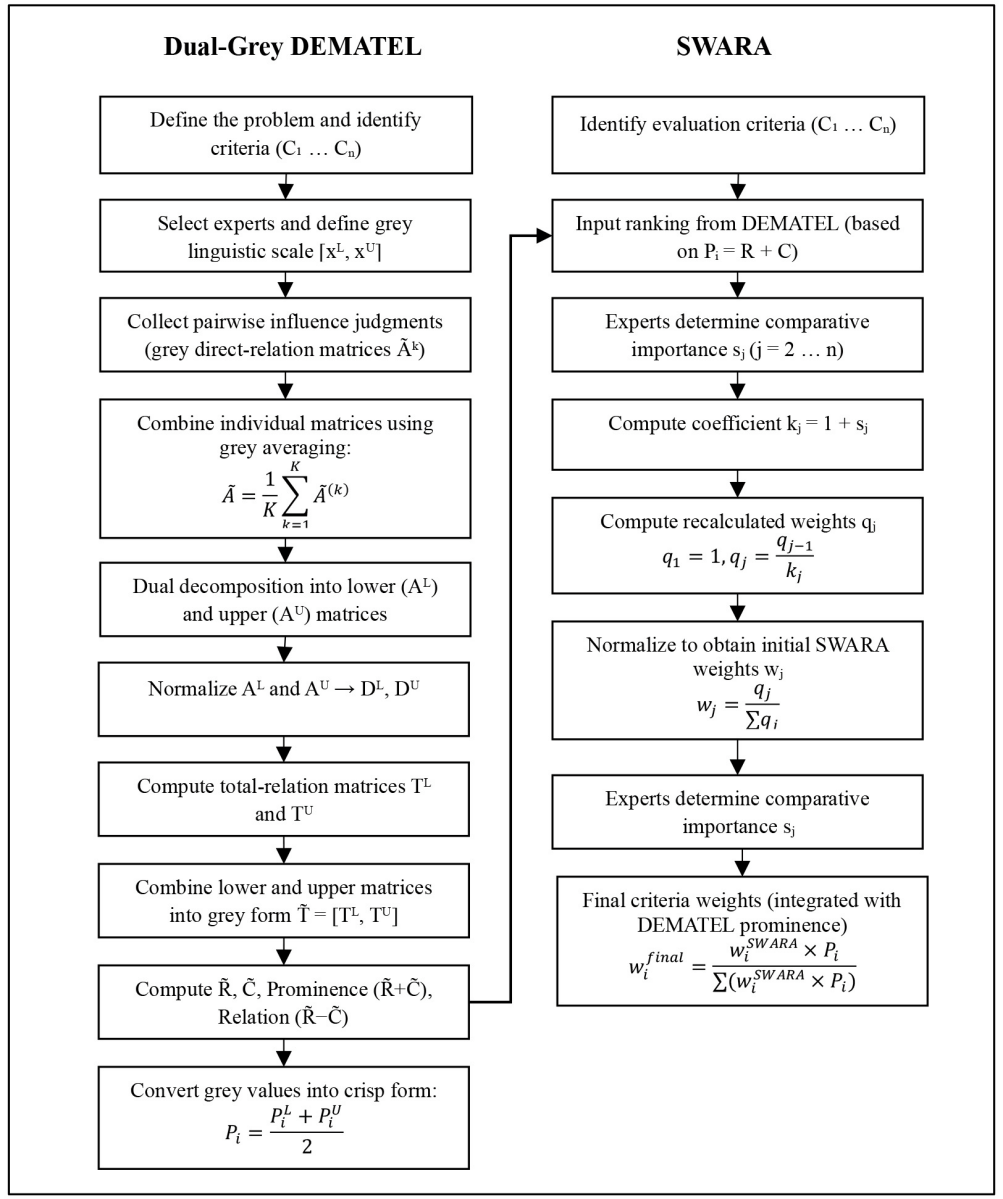
Research map.

This study adopts a structured multi-criteria decision-making (MCDM) approach to identify and prioritize the key drivers influencing cyber resilience in digital business ecosystems. Given the complexity and interdependence of cyber resilience capabilities, an integrated analytical framework combining Dual-Gray DEMATEL and Adaptive SWARA methods was employed to analyze the relationships among drivers and determine their relative importance. The research process was conducted in several sequential stages (Figure 1). First, a comprehensive literature review was performed to identify potential cyber resilience drivers reported in prior studies related to cybersecurity, organizational resilience, and digital ecosystem risk management. Based on this review, an initial list of resilience factors was compiled and subsequently refined through expert evaluation. Second, an expert panel consisting of specialists in cybersecurity, digital transformation, and information systems management was formed to validate the identified drivers and assess the relationships among them. A structured questionnaire was designed to collect expert judgments regarding the degree of influence among the resilience drivers. Third, the Dual-Gray DEMATEL method was applied to analyze the perceived influence structure among the drivers. The gray representation of expert judgments allows the model to capture uncertainty and imprecision inherent in human evaluations while preserving interval information during the analysis process. This step enables the identification of influential and influenced drivers within the cyber resilience system. Finally, the Adaptive SWARA method was used to determine the relative importance and priority of the identified drivers. The ordering of criteria in the SWARA process was informed by the prominence values obtained from the DEMATEL analysis, enabling a more structured prioritization of cyber resilience drivers.

### 3.2 Identification of drivers

The identification of cyber resilience drivers followed a two-stage process to ensure both theoretical comprehensiveness and practical relevance. First, a systematic literature review was conducted across major academic databases (Scopus, Web of Science, IEEE Xplore, and ScienceDirect) using keywords such as “cyber resilience,” “digital business ecosystem,” “threat intelligence,” and related terms. This review yielded an initial list of factors, which were then categorized and consolidated after removing duplicates and merging overlapping concepts. Second, the refined list was evaluated by a panel of ten experts from academia and industry with backgrounds in cybersecurity management, digital transformation, and organizational resilience. Experts assessed each factor for clarity, relevance, and appropriateness to digital business ecosystems. Through this evaluation, conceptually redundant or less relevant drivers were eliminated, resulting in a final set of 17 cyber resilience drivers (Table 3). These drivers span technological, human-cultural, and organizational-strategic domains, including AI-related capabilities such as C10 (Threat intelligence integration) and C13 (AI-driven monitoring).

**Table 3:** Taxonomy of cyber-resilience drivers.

Dimensions	Description	Criteria	Research study
Leadership and governance capabilities	Blend of directives, values, and orientations senior teams use to perform duties and make choices	Strategic risk assessment	Chandna and Tiwari, 2023
		Adaptive policy formulation	Moşteanu, 2020
		Compliance integration	Sukachova et al., 2025
		Stakeholder alignment	Tagarev and Yanakiev, 2020
		Crisis leadership	Kanaan et al., 2025
Organizational and Process Capabilities	Entity's skill to format and merge resources and competencies	Business continuity planning	Zaman et al., 2023
		Incident response orchestration	Annarelli et al., 2020
		Vendor risk oversight	Bagheri et al., 2023
		Adaptive process redesign	Urciuoli, 2015
Technological and Infrastructure Capabilities	Array of complex skills, knowledge, and actions converting resources into value yields	Threat intelligence integration	Gündüzyeli, 2025
		Network segmentation	Hausken, 2020
		Cloud security management	Tsai et al., 2022
		AI-driven monitoring	Sadeghi et al., 2024
Human and Cultural Capabilities	Degree to which staff are equipped to provide processes enabling swift, dependable, secure provisions	Awareness training programs	Saeed et al., 2023
		Cultural embedding	Loonam et al., 2020
		Behavioral risk mitigation	Gudla and Jamalpur, 2024
		Team collaboration enhancement	Annarelli and Palombi, 2021

### 3.3 Expert panel selection and validation

Expert selection followed purposive sampling based on two criteria: (1) minimum 5 years of professional experience in cybersecurity, information security, or digital risk management; and (2) direct involvement in implementing, governing, or auditing cybersecurity frameworks and digital infrastructures. The panel comprised 10 experts balanced between academia and industry to ensure both theoretical rigor and practical insight. To validate the expert evaluation process, inter-rater agreement was assessed using Fleiss' kappa, yielding a value of *K* = 0.73, indicating substantial agreement among experts. This validation step enhances the reliability of the collected judgments used in subsequent DEMATEL and SWARA analyses. Table 1 summarizes the demographic and professional characteristics of the participating experts.

### 3.4 Dual-Gray DEMATEL

To help non-specialist readers understand the logic of this method, it is useful to consider a simple analogy: imagine trying to understand which components of a complex security system affect one another. DEMATEL (Decision Making Trial and Evaluation Laboratory) creates a structured map of these perceived influences based on expert opinion. For instance, if experts believe that employee training strongly influences incident response speed, and incident response speed in turn affects business continuity, then investing in training could generate ripple effects throughout the entire resilience system. The “Gray” extension of DEMATEL acknowledges that experts rarely provide exact numerical judgments they are more comfortable expressing assessments such as “medium to high influence” rather than committing to a precise value. Gray numbers, expressed as intervals (e.g., [2, 3] for “Medium” influence), explicitly incorporate this uncertainty into the analysis rather than forcing artificial precision.

**Figure 2:**
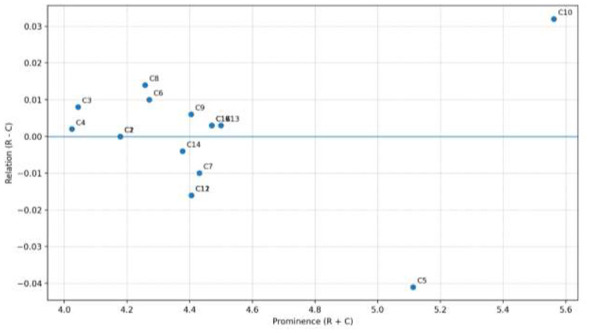
Prominence-relation map.

In applying this method, experts first assessed pairwise influences among the 17 cyber resilience drivers using a linguistic scale converted into gray interval numbers (Table 1). Individual judgments were aggregated to form the Initial Gray Direct-Relation Matrix (provided in full in Appendix A to preserve readability of the main text). This matrix was then normalized, and through successive matrix operations the Total Influence Matrix (T) was derived, capturing both direct and indirect influences among all drivers. From this matrix, two key metrics were calculated for each driver: prominence (*r*_*i*_+*c*_*i*_), representing the sum of influence exerted on others and received from others indicating overall centrality within the network; and relation (*r*_*i*_−*c*_*i*_), representing the net influence position. A positive relation value suggests the driver is a net influencer (perceived to affect others more than it is affected), while a negative value indicates a net influenced driver. These metrics were then used to construct an influence diagram (Figure 2), mapping the perceived structure of the cyber resilience system.

It is important to clarify the terminology used in this context. In DEMATEL methodology, the terms “cause” and “effect” refer specifically to a driver's perceived structural position within the influence network as judged by the expert panel they do not imply empirically proven, deterministic causal mechanisms. A driver identified as a “cause” (or net influencer) is simply one that experts perceive as exerting more influence on the system than it receives, suggesting it may serve as a hypothetical leverage point for interventions. The influence structure revealed by this analysis therefore represents the collective judgment of the expert panel and should be interpreted as a decision-support input rather than as evidence of actual, observable causal pathways.

### 3.5 Adaptive SWARA

To make the method accessible to non-specialist readers, consider this analogy: if an organization has limited resources and needs to decide which cyber resilience drivers to invest in first, SWARA (Step-wise Weight Assessment Ratio Analysis) provides a structured way to make that decision. Experts first rank all drivers from most to least important. Then, they compare each driver with the next one on the list, asking: “How much more important is Driver X compared to Driver Y?” The “Adaptive” modification integrates the results from the DEMATEL analysis specifically, the prominence values that indicate each driver's centrality in the influence network. This ensures that the final importance weights reflect not only expert preference but also how drivers interact within the system, creating a more holistic prioritization.

In practice, experts ranked the 17 cyber resilience drivers based on their perceived importance for enhancing resilience in digital business networks. For each consecutive pair in this ranking, experts then provided a relative importance ratio (*s*_*j*_), expressing how much more important the higher-ranked driver is compared to the next one. These ratios were used to sequentially calculate the comparative coefficient (*k*_*j*_), recalculated weight (*w*_*j*_), and finally the normalized weight (*q*_*j*_) for each driver. Crucially, the prominence score (*r*_*i*_+*c*_*i*_) from the DEMATEL analysis was incorporated as a weighting factor during this process, making the SWARA method “adaptive” to the systemic influence structure. The resulting normalized weights yield the final priority ranking, identifying which drivers are deemed most critical for strategic investment.

It is essential to clarify the nature and limitations of these weights. The priority weights derived from Adaptive SWARA represent the collective perceptions and judgments of the expert panel regarding the relative importance of different resilience drivers. They are not based on observed organizational performance data or empirically verified impact measurements. Consequently, this prioritization should be interpreted as a decision-support framework that provides structured guidance based on expert insight, rather than as definitive evidence of actual effectiveness. Organizations applying these results should consider them alongside contextual factors such as implementation feasibility, resource constraints, and specific organizational vulnerabilities.

### 3.6 Integrated analytical framework and limitations

The integrated framework synthesizes insights from both methodological components to provide a comprehensive assessment of cyber resilience drivers. Dual-Gray DEMATEL identifies which drivers function as central influencers within the resilience network, highlighting potential leverage points where interventions might generate cascading effects. Adaptive SWARA determines which drivers hold the highest strategic importance, offering guidance for resource allocation under constraints. Together, they produce a multi-faceted ranking that balances systemic influence with perceived priority, supporting more nuanced and resilient decision-making for digital business networks.

Several important limitations must be acknowledged regarding this expert-based approach. First, the derived influence structure and priority weights reflect the collective perceptions and judgments of the expert panel at a specific point in time. They represent hypothetical leverage points rather than empirically verified causal mechanisms observed in organizational settings. Second, while the gray-number representation captures uncertainty in expert assessments, it does not eliminate the subjectivity inherent in qualitative judgments. Third, the framework assumes that the identified drivers are sufficiently comprehensive and that their interdependencies remain stable across different organizational contexts assumptions that may not hold in all cases. Fourth, the expert panel, while carefully selected, represents a limited sample, and perceptions may vary across industries, regions, or organizational sizes.

Consequently, this integrated framework should be interpreted primarily as a structured decision-support tool rather than as direct evidence of actual organizational impacts. Its value lies in providing a systematic and transparent basis for strategic discussions about cyber resilience investments. Practitioners applying these results are advised to complement them with organizational-specific data, implementation feasibility assessments, and continuous monitoring of evolving threat landscapes.

### 3.7 Questionnaire design and data collection

A structured questionnaire was developed to collect expert judgments regarding the interrelationships among the identified cyber resilience drivers. The questionnaire was constructed based on the validated criteria obtained through the literature review and the expert screening process described in the previous section. Its design specifically supported the implementation of the Dual Gray DEMATEL and Adaptive SWARA methods by enabling experts to express their evaluations under conditions of uncertainty. The questionnaire consisted of three main sections. The first section collected demographic and professional information about the participating experts, including their organizational role, years of professional experience, and area of specialization. The second section presented concise operational definitions of the identified cyber resilience drivers in order to ensure a consistent conceptual understanding among all respondents. The third section contained pairwise influence assessment matrices in which experts evaluated the degree to which each driver influences another driver within the cyber resilience system (see Appendix A).

Considering the ambiguity and subjectivity associated with evaluating complex cybersecurity relationships, a Gray linguistic evaluation scale was employed. Experts expressed their judgments using predefined linguistic terms, which were subsequently converted into Gray interval numbers for computational analysis. The Gray representation allows the uncertainty inherent in expert opinions to be explicitly captured while maintaining the flexibility of linguistic assessments. The linguistic scale used in this study is presented in Table 2.

Each expert evaluated the influence of driver *i* on driver *j* using the linguistic scale, resulting in an individual gray pairwise comparison matrix. These matrices were subsequently aggregated to construct the initial gray direct-relation matrix required for the Dual Gray DEMATEL analysis. The aggregation process was conducted by combining the lower and upper bounds of the gray intervals across experts to obtain a collective representation of the panel's judgments. The questionnaire was distributed electronically to the selected expert panel between March 2025 and May 2025. Prior to the final data collection stage, the questionnaire was pilot tested with a subset of experts to evaluate clarity, consistency, and interpretability. Based on their feedback, minor revisions were applied to improve wording precision and reduce potential ambiguity. To enhance methodological transparency and reproducibility, the complete expert evaluation questionnaire including expert profile forms, driver definitions, pairwise assessment matrices, and sample evaluation items is provided in Appendix A. This Supplementary material allows future researchers to replicate the data collection process and apply the proposed framework in different organizational or industrial contexts.

### 3.8 Dual-Gray DEMATEL method

To examine the complex interrelationships among cyber resilience drivers within digital business ecosystems, this study employed the Dual-Gray DEMATEL method. DEMATEL (Decision Making Trial and Evaluation Laboratory) is a widely used multi-criteria decision-making technique developed to analyze and visualize the influence structure among interdependent factors in complex systems. However, conventional DEMATEL approaches typically rely on precise numerical evaluations, which may not adequately represent the uncertainty and ambiguity associated with expert judgments in cybersecurity and digital business contexts characterized by evolving threats, incomplete information, and subjective assessments. To address this limitation, the present study integrates Gray Systems Theory with the DEMATEL framework. Gray theory enables uncertain expert evaluations to be expressed as interval values rather than fixed numerical scores, thereby providing a more realistic representation of linguistic assessments under uncertainty. Unlike conventional DEMATEL implementations based on crisp numerical judgments, the proposed Dual Gray DEMATEL framework preserves both lower and upper uncertainty bounds throughout the entire computational process. By maintaining the interval structure of expert evaluations during matrix construction, normalization, and total-relation analysis, the method provides a more robust representation of ambiguous expert perceptions. This characteristic is particularly important in cybersecurity and digital business environments where decision conditions are inherently uncertain and continuously evolving.

In this study, expert evaluations were collected using Gray linguistic variables corresponding to interval numbers (see Table 2 in Section 3.4). The collected judgments were subsequently transformed into Dual-Gray direct-relation matrices representing the lower and upper bounds of influence among the identified cyber resilience drivers. The aggregated matrices were then analyzed using the Dual-Gray DEMATEL procedure, which was implemented through five main stages: (1) construction of the initial direct-relation matrices, (2) matrix normalization, (3) derivation of the total-relation matrix, (4) computation of prominence and relation indices, and (5) visualization of the perceived influence structure among the drivers.

*Step 1. Construction of the Gray direct-relation matrix*

Based on the pairwise evaluations provided by the expert panel, the initial grey direct relation matrix was constructed, as shown in Equation (1):


$$\begin{array}{c} \tilde{Z}=\left[\begin{array}{c} \left[z_{11}^{L},z_{11}^{U}\right]\cdots \left[z_{1n}^{L},z_{1n}^{U}\right]\\ \vdots \ddots \vdots \\ \left[z_{n1}^{L},z_{n1}^{U}\right]\cdots \left[z_{nn}^{L},z_{nn}^{U}\right] \end{array}\right] \end{array}$$ (1)

where:

- *z*^*L*^and *z*^*U*^denote the lower and upper bounds of the Gray interval,
- *n*represents the number of evaluation criteria,
- and each matrix element indicates the degree of influence of one driver on another.

The individual expert matrices were aggregated using arithmetic averaging to obtain the collective Gray direct-relation matrix. The aggregated Gray direct-relation matrices for both lower bound (*A*^*L*^) and upper bound (*A*^*U*^) values are presented in Appendix B (Tables 4, 5, respectively).

**Table 4:** Dual-Gray row and column sums of the total-relation matrices (*T*^*L*^, *T*^*U*^).

Criterion	(Row sum of T^L^)	(Row sum of T^U^)	(Col sum of T^L^)	(Col sum of T^U^)
C1	1.768	2.410	1.767	2.411
C2	1.768	2.410	1.767	2.411
C3	1.714	2.338	1.702	2.335
C4	1.703	2.323	1.696	2.325
C5	2.233	2.839	2.311	2.843
C6	1.806	2.475	1.795	2.466
C7	1.870	2.551	1.875	2.565
C8	1.800	2.472	1.785	2.458
C9	1.864	2.546	1.857	2.540
C10	2.473	3.121	2.439	3.091
C11	1.854	2.535	1.868	2.552
C12	1.854	2.535	1.868	2.552
C13	1.902	2.601	1.896	2.601
C14	1.849	2.525	1.850	2.531
C15	1.889	2.584	1.883	2.584
C16	1.889	2.584	1.883	2.584
C17	1.889	2.584	1.883	2.584

**Table 5:** Initial ranking of cyber-resilience drivers based on DEMATEL prominence.

Rank	Driver code	Cyber-resilience driver	Prominence value (P_i_=R_i_+C_i_)
1	C10	Threat intelligence integration	5.562
2	C5	Crisis leadership	5.113
3	C13	AI-driven monitoring	4.500
4	C15	Cultural embedding of cyber-resilience	4.470
4	C16	Behavioral risk mitigation	4.470
4	C17	Team collaboration enhancement	4.470
5	C7	Incident response orchestration	4.431
6	C11	Network segmentation	4.405
6	C12	Cloud security management	4.405
7	C9	Adaptive process redesign	4.404
8	C14	Awareness and training programs	4.377
9	C6	Business continuity planning	4.271
10	C8	Vendor risk oversight	4.258
11	C1	Strategic risk assessment	4.178
11	C2	Adaptive policy formulation	4.178
12	C3	Compliance integration	4.044
13	C4	Stakeholder alignment	4.024

*Step 2. Normalization of the Gray direct-relation matrix*

To ensure the comparability of influence intensities and satisfy the convergence requirement of the DEMATEL methodology, the aggregated dual-gray direct-relation matrices were normalized. Since the proposed framework preserves uncertainty through interval gray numbers, the normalization procedure was conducted separately for the lower-bound and upper-bound matrices. Following the Dual-Gray DEMATEL approach, the normalization coefficient was determined based on the maximum row sum of the upper-bound matrix, as given in Equation (2), to provide a conservative and computationally stable scaling factor.


$$\begin{array}{c} s=\frac{1}{\underset{i}{\max}\sum_{j=1}^{n}a_{ij}^{U}} \end{array}$$ (2)

where $a_{ij}^{U}$denotes the upper bound of the aggregated gray influence of criterion *i* on criterion *j*. The upper-bound row sum was adopted to determine the normalization coefficient because it represents the maximum potential influence intensity within the system. As indicated in Equation (2), using the upper bound provides a conservative scaling mechanism that ensures the convergence condition of the DEMATEL model while preserving the uncertainty embedded in the grey evaluations. Based on the obtained scaling coefficient, the normalized lower-bound and upper-bound matrices were then calculated as shown in Equations (3) and (4), respectively:


$$\begin{array}{c} D^{L}=s^{L}A^{L} \end{array}$$ (3)

$$\begin{array}{c} D^{U}=s^{U}A^{U} \end{array}$$ (4)

where:

- *A*^*L*^ and *A*^*U*^represent the aggregated lower-bound and upper-bound direct-relation matrices, respectively;
- *D*^*L*^ and *D*^*U*^ denote the corresponding normalized matrices;
- *s*^*L*^ and *s*^*U*^ are the normalization coefficients associated with the lower and upper bounds.

This normalization process constrains all matrix elements to the interval [0, 1], thereby ensuring numerical stability and preventing disproportionate propagation of influence during the subsequent computation of the total-relation matrices. Moreover, by preserving both lower and upper influence boundaries throughout the normalization stage, the uncertainty embedded in expert judgments is retained within the causal analysis process. In this study, the maximum row sums of the aggregated lower-bound and upper-bound matrices were identified as 8.50 and 12.50, respectively. Accordingly, the normalization coefficients were obtained as:


$$\begin{array}{c} s^{L}=\frac{1}{8.50}=0.1176 \end{array}$$

$$\begin{array}{c} s^{U}=\frac{1}{12.50}=0.0800 \end{array}$$

These coefficients were subsequently used to derive the normalized lower bound and upper bound matrices, which are provided in Appendix B (Tables B1, B2).

*Step 3: Construction of the total-relation matrices*

After obtaining the normalized gray direct-relation matrices (*D*^*L*^) and (*D*^*U*^), the total relation matrices were computed to capture both the direct and indirect interdependencies among the cyber resilience drivers. In the DEMATEL framework, the normalized matrices reflect only the immediate influence among factors, whereas the total relation matrices reveal the cumulative effects propagated throughout the system. Accordingly, the lower-bound and upper-bound total relation matrices were calculated as shown in Equations (5) and (6), respectively:


$$\begin{array}{c} \begin{array}{c} T^{L}=D^{L}{\left(I-D^{L}\right)}^{-1} \end{array} \end{array}$$ (5)

$$\begin{array}{c} \begin{array}{c} T^{U}=D^{U}{\left(I-D^{U}\right)}^{-1} \end{array} \end{array}$$ (6)

where *I* represents the identity matrix with a dimension of 17 × 17. The matrices *T*^*L*^ and *T*^*U*^, respectively, denote the lower and upper bounds of the total influence levels among the evaluated drivers. These matrices incorporate both the direct effects between drivers and the indirect effects transmitted through other drivers in the network. Therefore, the total relation matrices provide a comprehensive representation of the perceived influence structure and interaction intensity within the cyber resilience system. The calculated lower and upper total relation matrices are presented in Appendix B (Tables B3, B4).

*Step 4: Determination of prominence and relation values*

After deriving the total-relation matrices, the prominence and relation indices of the cyber-resilience drivers were determined in order to identify their overall importance and causal roles within the system. The analysis was conducted based on the total-relation matrix *T*. In the Dual-Gray DEMATEL framework, these calculations were performed separately for the lower and upper total-relation matrices (*T*^*L*^, *T*^*U*^), thereby preserving the uncertainty structure throughout the evaluation process. For each driver *i*, the row sum and column sum were computed as shown in Equations (7) and (8), respectively:

The vectors *R* and *C* were calculated as follows:


$$\begin{array}{c} R_{i}=\overset{n}{\underset{j=1}{\sum }}t_{ij} \end{array}$$ (7)

$$\begin{array}{c} C_{i}=\overset{n}{\underset{j=1}{\sum }}t_{ji} \end{array}$$ (8)

where *R*_*i*_ denotes the sum of the *i*-th row and reflects the total influence exerted by driver *i*, whereas *C*_*i*_ denotes the sum of the *i*-th column and represents the total influence received by driver *i*. Based on these values, the prominence and relation indices were then obtained as presented in Equations (9) and (10), respectively:


$$\begin{array}{c} R_{i}+C_{i} \end{array}$$ (9)

$$\begin{array}{c} R_{i}-C_{i} \end{array}$$ (10)

The prominence value (*R*_*i*_+*C*_*i*_)reflects the overall degree of interaction of each driver within the system. Drivers with higher prominence values are considered more critical, as they are strongly connected to other drivers through both outgoing and incoming influences. The relation value (*R*_*i*_−*C*_*i*_)determines the causal role of each driver. If (*R*_*i*_−*C*_*i*_)>0, the driver belongs to the cause group, meaning it predominantly influences other drivers. Conversely, if (*R*_*i*_−*C*_*i*_) < 0, the driver belongs to the effect group and is primarily influenced by others. The computed prominence and relation values are presented in Table 4. Table 4 reports the Dual-Gray row and column sums of the total-relation matrices (*T*^*L*^, *T*^*U*^), which correspond to the outgoing (R) and incoming (C) influence intervals for each driver. Based on the computed prominence and relation values, a Prominence-Relation Map was constructed to visualize the causal relationships among the cyber resilience drivers (Figure 2).

*Step 5. Causal analysis and visualization*

Based on the computed prominence and relation values, a Prominence-Relation Map was constructed to visualize the structural relationships among the identified cyber resilience drivers (Figure 2). In this map, the horizontal axis (*R*_*i*_+*C*_*i*_)represents the prominence of each driver, indicating its overall degree of involvement within the system, while the vertical axis (*R*_*i*_−*C*_*i*_)represents the net causal effect. Drivers with positive (*R*_*i*_−*C*_*i*_)values are categorized as cause factors, meaning that they exert greater influence on other drivers than they receive. In contrast, drivers with negative (*R*_*i*_−*C*_*i*_)values are classified as effect factors, indicating that they are primarily influenced by other elements within the system. The Prominence-Relation Map provides a comprehensive visualization of the interaction structure among cyber resilience drivers and facilitates the identification of the most influential causal factors for managerial prioritization and strategic decision-making.

The results obtained from the Dual Gray DEMATEL analysis provide valuable insights into the structural relationships among the identified cyber-resilience drivers. In particular, the prominence values derived from the total-relation matrix reflect the overall involvement of each driver within the system by capturing both the influence it exerts on other drivers and the influence it receives. While DEMATEL is primarily used to analyze causal interdependencies among factors, it does not directly provide normalized priority weights. Therefore, to translate the structural influence information obtained from DEMATEL into quantitative importance weights, the Adaptive SWARA method is employed in the subsequent stage of the analysis. By using the DEMATEL-derived prominence values as the basis for the initial ranking of the drivers, the SWARA procedure incorporates the systemic influence structure into the weighting process, thereby providing a more structured and informed prioritization of cyber-resilience drivers.

### 3.9 Adaptive SWARA method

Following the identification of the prominence and influence structure of cyber-resilience drivers through the Dual Gray DEMATEL analysis, the present study employed the Adaptive SWARA (Step-wise Weight Assessment Ratio Analysis) method to determine the final weights of the drivers. The SWARA method is a well-known multi-criteria decision-making technique designed to derive criterion weights through a stepwise evaluation of their relative importance. In conventional SWARA applications, the initial ordering of criteria is typically obtained directly from expert rankings. However, such ordering may remain highly subjective and may not fully reflect the structural relationships among criteria. To address this limitation, the adaptive version of SWARA applied in this study integrates the results of the Dual Gray DEMATEL analysis into the weighting procedure. Specifically, the prominence values derived from the DEMATEL total-relation matrix are used to establish the initial ranking of the cyber-resilience drivers. By incorporating the systemic influence structure revealed by DEMATEL, the Adaptive SWARA approach provides a more structurally informed weighting mechanism compared with conventional SWARA implementations that rely solely on subjective expert ordering. As illustrated in Figure 1 (Research Framework), the Adaptive SWARA method represents the final stage of the proposed methodological framework and converts the DEMATEL-derived prominence ordering into normalized criterion weights. The Adaptive SWARA procedure applied in this study consists of five main steps, described as follows.

*Step 1: Initial ranking of cyber-resilience drivers based on DEMATEL prominence*

In the first step, the cyber-resilience drivers are ranked according to their prominence values obtained from the Dual Gray DEMATEL analysis. The prominence value represents the overall importance of each driver within the system by considering both the influence it exerts on other drivers and the influence it receives from them. As shown in Equation (11), the prominence value for each driver was calculated as follows:


$$\begin{array}{c} P_{i}=R_{i}+C_{i} \end{array}$$ (11)

where *R*_*i*_ denotes the sum of the *i*-th row of the total-relation matrix, representing the total influence dispatched by driver *i* to other drivers, and *C*_*i*_represents the sum of the *i*-th column, indicating the total influence received by driver *i*. The prominence value *P*_*i*_, therefore, reflects the overall involvement of each driver in the cyber-resilience system. Based on these values, the drivers are arranged in descending order to establish the initial ranking used in the Adaptive SWARA procedure. To obtain a single ranking index for this step, the lower and upper prominence values derived from the dual gray matrices were aggregated using the arithmetic mean. The resulting ordered list of drivers is presented in Table 5.

Based on this initial ordering, the comparative importance of each driver relative to the preceding driver is determined in the next step of the Adaptive SWARA method.

*Step 2: Determination of the comparative importance coefficient*

After establishing the initial ranking of the cyber-resilience drivers based on their DEMATEL prominence values, the next step of the Adaptive SWARA method involves determining the comparative importance coefficient. This coefficient reflects the relative importance of each driver compared with the preceding driver in the ranked list. Starting from the second driver in the ordered sequence, experts assess the comparative importance of driver *j* with respect to the previous driver (*j*−1). This assessment is expressed through the comparative importance coefficient *s*_*j*_, which indicates how much less important driver *j* is relative to the preceding driver. A larger value of *s*_*j*_ implies a greater decrease in importance compared with the previous driver. The comparative importance coefficient is calculated as shown in Equation (12):


$$\begin{array}{c} \begin{array}{c} s_{j}=\frac{1}{K}\sum_{k=1}^{K}s_{j}^{\left(k\right)},j=2,\ldots ,n \end{array} \end{array}$$ (12)

*s*_*j*_: comparative importance coefficient of criterion *j*,

$s_{j}^{\left(k\right)}$: assessment provided by expert *k*for criterion *j*,

*K*: number of experts,

*n*: total number of criteria.

where *s*_*j*_ represents the relative importance of driver *j* compared to driver (*j*−1) in the ordered list. For the first driver in the ranking, no comparison is required, and, therefore, *s*_1_ = 0. The calculated comparative importance coefficients for the cyber-resilience drivers are presented in Table 6, which forms the basis for determining the adjustment coefficients in the subsequent step of the Adaptive SWARA procedure.

**Table 6:** Comparative importance coefficients (*s*_*j*_)for SWARA.

Order *j*	Criterion	Driver	P_crisp	s_j (vs. previous)
1	C10	Threat intelligence integration	5.562	
2	C5	Crisis leadership	5.113	0.10
3	C13	AI-driven monitoring	4.500	0.12
4	C15	Cultural embedding of cyber-resilience	4.470	0.05
5	C16	Behavioral risk mitigation	4.470	0.00
6	C17	Team collaboration enhancement	4.470	0.00
7	C7	Incident response orchestration	4.431	0.08
8	C11	Network segmentation	4.405	0.04
9	C12	Cloud security management	4.405	0.00
10	C9	Adaptive process redesign	4.404	0.01
11	C14	Awareness and training programs	4.377	0.03
12	C6	Business continuity planning	4.271	0.06
13	C8	Vendor risk oversight	4.258	0.02
14	C1	Strategic risk assessment	4.178	0.07
15	C2	Adaptive policy formulation	4.178	0.00
16	C3	Compliance integration	4.044	0.15
17	C4	Stakeholder alignment	4.024	0.05

*Step 3: Determination of adjustment coefficients*

Following the calculation of the comparative importance coefficients, the adjustment coefficients are determined to reflect the relative significance of each cyber-resilience driver within the Adaptive SWARA framework. The adjustment coefficient modifies the importance of each criterion according to its comparative importance relative to the preceding criterion in the ranked sequence. As shown in Equation (13), the adjustment coefficient for each driver was calculated as follows:


$$k_{j}=\{\begin{array}{c} 1,j=1\\ s_{j}+1,j>1 \end{array}$$ (13)

where *k*_*j*_ denotes the adjustment coefficient of criterion *j*, and *s*_*j*_ represents the comparative importance coefficient obtained in the previous step. For the first-ranked criterion, the adjustment coefficient is assigned a value of 1 because no prior comparison exists. Using the obtained adjustment coefficients, the recalculated weights of the cyber-resilience drivers can subsequently be determined. The calculated adjustment coefficients for all criteria are presented in Table 7.

**Table 7:** Adjustment coefficients (*k*_*j*_)and recalculated weights (*q*_*j*_).

Order j	Criterion	s_j_	k_j_ = 1+s_j_	q_j_
1	C10			1.000000
2	C5	0.10	1.10	0.909091
3	C13	0.12	1.12	0.811688
4	C15	0.05	1.05	0.772084
5	C16	0.00	1.00	0.772084
6	C17	0.00	1.00	0.772084
7	C7	0.08	1.08	0.714893
8	C11	0.04	1.04	0.687397
9	C12	0.00	1.00	0.687397
10	C9	0.01	1.01	0.680591
11	C14	0.03	1.03	0.660768
12	C6	0.06	1.06	0.623366
13	C8	0.02	1.02	0.611143
14	C1	0.07	1.07	0.571162
15	C2	0.00	1.00	0.571162
16	C3	0.15	1.15	0.496663
17	C4	0.05	1.05	0.472060

*Step 4: Calculation of recalculated weights*

After determining the adjustment coefficients, the recalculated weights of the cyber-resilience drivers are obtained sequentially. These weights reflect the relative importance of each driver after incorporating the comparative significance adjustments derived from the previous step. As presented in Equation (14), the recalculated weight for each criterion was computed recursively as follows:


$$q_{j}=\left\{ \begin{array}{l} 1,j=1\\ \frac{q_{j-1}}{k_{j}},j>1 \end{array}\right.$$ (14)

where *q*_*j*_ represents the recalculated weight of criterion *j*, and *k*_*j*_ denotes the corresponding adjustment coefficient. The first-ranked criterion is assigned an initial value of *q*_1_ = 1, while the weights of the remaining criteria are determined progressively according to their relative importance. The resulting recalculated weights for all cyber-resilience drivers are reported in Table 8, which provides the basis for deriving the final normalized weights in the subsequent step of the Adaptive SWARA method.

**Table 8:** Final SWARA weights (*w*_*j*_).

Final rank	Criterion	Driver	q_j_	w_j_
1	C10	Threat intelligence integration	1.000000	0.084648
2	C5	Crisis leadership	0.909091	0.076953
3	C13	AI-driven monitoring	0.811688	0.068708
4	C15	Cultural embedding of cyber-resilience	0.772084	0.065355
4	C16	Behavioral risk mitigation	0.772084	0.065355
4	C17	Team collaboration enhancement	0.772084	0.065355
5	C7	Incident response orchestration	0.714893	0.060514
6	C11	Network segmentation	0.687397	0.058187
6	C12	Cloud security management	0.687397	0.058187
7	C9	Adaptive process redesign	0.680591	0.057611
8	C14	Awareness and training programs	0.660768	0.055933
9	C6	Business continuity planning	0.623366	0.052767
10	C8	Vendor risk oversight	0.611143	0.051732
11	C1	Strategic risk assessment	0.571162	0.048348
11	C2	Adaptive policy formulation	0.571162	0.048348
12	C3	Compliance integration	0.496663	0.042042
13	C4	Stakeholder alignment	0.472060	0.039959

*Step 5: Normalization and derivation of final SWARA weights*

In the final stage of the Adaptive SWARA method, the recalculated weights are normalized in order to derive the final relative weights of the cyber-resilience drivers. This normalization process ensures that the sum of all weights equals one, thereby enabling a meaningful comparison of the relative importance of the identified drivers. As shown in Equation (15), the final normalized weight of each criterion was calculated as follows:


$$\begin{array}{c} \begin{array}{c} w_{j}=\frac{q_{j}}{\sum_{j=1}^{n}q_{j}} \end{array} \end{array}$$ (15)

where *w*_*j*_ denotes the final normalized weight of criterion *j*, *q*_*j*_ represents the recalculated weight obtained from the previous step, and *n* is the total number of cyber-resilience drivers considered in the analysis. The resulting weights indicate the overall priority and relative contribution of each driver to cyber resilience enhancement in the healthcare supply chain. Since the initial ranking was derived from the prominence values obtained through the Dual-Gray DEMATEL analysis, the Adaptive SWARA approach provides a more objective and structurally informed weighting mechanism compared with conventional SWARA applications that rely solely on subjective expert ordering. The final normalized weights and ranking results of the cyber-resilience drivers are presented in Table 8.

## 4 Results and discussion

The results of this study provide insights into the perceived structural relationships and relative importance of cyber resilience drivers in AI-enabled digital business networks. By integrating Dual-Gray DEMATEL and Adaptive SWARA, the analysis captures both the perceived interdependency structure among the identified drivers and their prioritization under conditions of uncertainty. Prior studies emphasize that cyber resilience is not a single technological capability but rather a multidimensional organizational capacity involving governance, technological readiness, and adaptive management mechanisms (Annarelli et al., 2020; Bagheri et al., 2023). Consistent with this view, the DEMATEL results suggest that the examined drivers may interact within a complex influence network rather than functioning as independent resilience mechanisms.

The prominence-relation analysis, summarized in Table 4, highlights that some drivers appear to exert comparatively stronger perceived influence on other elements of the cyber resilience system, while others seem more dependent on surrounding organizational and technological conditions. Such systemic interaction patterns align with previous research suggesting that resilience within digitally interconnected environments may emerge from coordinated interactions between organizational capabilities, cybersecurity governance, and technological infrastructures (Hausken, 2020; Loonam et al., 2020). The perceived influence structure identified in this study therefore reflects expert assessments of how resilience capabilities might evolve within digital ecosystems characterized by high interconnectivity and shared digital infrastructures (Tsai et al., 2022; Zhu et al., 2020).

The Adaptive SWARA results, presented in Table 5, further reveal variations in the perceived strategic importance of the cyber resilience drivers. While certain drivers receive relatively higher weights, several drivers display close weight values, indicating that cyber resilience in AI-enabled business ecosystems may depend on a combination of complementary capabilities rather than a single dominant factor. This finding is consistent with prior studies emphasizing that effective cyber resilience strategies could require integrated managerial, technological, and organizational measures to address increasingly complex cyber threats (Chandna and Tiwari, 2023; Saeed et al., 2023). Consequently, the obtained rankings, consolidated in Table 6, should be interpreted as indicative prioritization patterns derived from expert evaluations rather than deterministic or universally generalizable rankings. As discussed in Section 3.6, this framework functions as a structured decision-support tool and does not constitute direct evidence of empirically observed organizational impacts.

### 4.1 Theoretical implications

This study contributes to the literature on cyber resilience and digital ecosystems in several ways. First, it extends existing research by conceptualizing cyber resilience within AI-enabled digital business networks as a systemic capability shaped by interconnected organizational and technological drivers. Previous research has emphasized the importance of resilience as an organizational capability enabling firms to anticipate, absorb, and adapt to cyber disruptions (Barasa et al., 2018; Urciuoli, 2015). The findings of this study reinforce this perspective by demonstrating that cyber-resilience drivers operate within an interdependent influence structure rather than as isolated organizational factors.

Second, the study contributes methodologically by integrating Dual-Gray DEMATEL with Adaptive SWARA to address two critical challenges frequently observed in cyber-resilience evaluation studies: uncertainty in expert judgments and interdependency among evaluation criteria. Gray-based approaches allow decision models to capture ambiguity and incomplete information, which are common in emerging digital environments where precise quantitative data are often unavailable (Govindan et al., 2016; Rajesh and Ravi, 2017). By incorporating gray numbers into the DEMATEL procedure, the proposed framework improves the representation of uncertainty in expert assessments.

Third, the study advances multi-criteria decision-making research by introducing a structurally informed weighting procedure. Unlike conventional SWARA implementations that rely solely on subjective expert ranking, the proposed Adaptive SWARA approach incorporates prominence information obtained from the DEMATEL analysis as the initial ranking basis. This integration enables the weighting process to reflect not only perceived importance but also the perceived structural role of each driver within the cyber-resilience influence network. Similar hybrid decision-making frameworks have recently been applied to complex cybersecurity and digital transformation problems where multiple interacting criteria must be evaluated simultaneously (Kara et al., 2025; Sadeghi et al., 2024). It should be noted, however, that the resulting weights represent expert-derived prioritization patterns, as discussed in Section 3.6.

### 4.2 Practical implications

From a managerial perspective, the findings provide practical guidance for organizations seeking to strengthen cyber resilience within AI-enabled digital ecosystems. As digital transformation increases the level of interconnection among firms, platforms, and supply chain partners, organizations become more exposed to cyber risks that may propagate across digital networks (Tagarev and Yanakiev, 2020; Zaman et al., 2023). The prioritization results obtained in this study may therefore assist managers in identifying the drivers that require greater strategic attention when designing cyber-resilience initiatives.

Drivers identified as net influencers in the DEMATEL analysis may represent potential leverage points that could influence multiple aspects of organizational resilience. Focusing on these drivers may help organizations enhance the effectiveness of cybersecurity governance, technological preparedness, and organizational response capabilities. Previous research suggests that proactive cyber-resilience strategies require coordinated investments in technological infrastructure, organizational processes, and risk management mechanisms (Annarelli and Palombi, 2021; Kanaan et al., 2025).

In addition, the proposed analytical framework offers decision-makers a structured tool for evaluating cyber-resilience priorities under conditions of uncertainty. Since many cyber-resilience challenges involve qualitative assessments and expert knowledge rather than purely quantitative data, gray-based decision-making approaches provide practical support for strategic planning in uncertain environments (Asnaashari et al., 2023; Govindan et al., 2016). Consequently, the integrated Dual-Gray DEMATEL-Adaptive SWARA framework may support organizations in allocating resources more effectively and designing balanced resilience strategies across interconnected digital operations. However, as emphasized in Section 3.6, these prioritization results should be interpreted as indicative guidance derived from expert perceptions and should be complemented with context-specific organizational assessments.

### 4.3 Limitations and future research

Despite its contributions, this study has several limitations that should be acknowledged. First, the findings are derived from expert evaluations and, therefore, reflect perceived influence structures within the contextual scope of the selected expert panel. Although the expert selection process and consistency assessment were carefully conducted (Section 3.6), different expert groups or industry contexts may lead to alternative prioritization outcomes. Second, the study adopts an exploratory expert-based multi-criteria decision-making approach and, therefore, does not establish empirically validated causal relationships among the identified cyber-resilience drivers. The DEMATEL results represent perceived influence patterns rather than definitive causal effects. Consequently, the findings should be interpreted with appropriate caution, as elaborated in Section 3.6.

Third, although the gray-based approach improves the representation of uncertainty in expert judgments, the framework remains dependent on subjective evaluations. Future studies may complement the proposed approach by incorporating empirical datasets, quantitative validation techniques, or longitudinal analyses to examine how cyber-resilience drivers influence organizational performance over time. Future research may also extend this study by applying the proposed framework to different industries, organizational sizes, or national contexts in order to evaluate the generalizability of the results. Comparative analyses involving alternative multi-criteria decision-making methods may further enhance the understanding of cyber-resilience prioritization in AI-enabled digital ecosystems.

## 5 Conclusion

As digital business networks become increasingly interconnected and reliant on artificial intelligence, organizations face growing challenges in maintaining operational continuity in the face of cyber disruptions. This study set out to identify and prioritize the key drivers that contribute to cyber resilience within AI-enabled digital ecosystems. Applying an integrated multi-criteria decision-making framework that combines Dual-Gray DEMATEL and Adaptive SWARA, the research moves beyond simple factor listing toward a structured understanding of the perceived influence relationships and relative importance of 17 cyber-resilience drivers.

The analysis reveals that Threat Intelligence Integration (C10) emerged as the highest-priority driver, suggesting that the systematic collection, analysis, and operationalization of threat data is perceived by experts as the most critical enabler of cyber resilience in digital business networks. This was followed by Strategic Risk Assessment (C5) and AI-driven Monitoring (C13), indicating that adaptive governance mechanisms and intelligent detection capabilities collectively form the backbone of a resilient digital infrastructure. The DEMATEL analysis further demonstrated that drivers such as C10 and C5 exhibit positive net-influencer profiles, meaning they are perceived as foundational drivers that may exert influence on multiple downstream resilience capabilities. Conversely, drivers with negative net-receiver profiles such as those related to incident recovery and business continuity are more likely to be shaped by improvements in the influencer drivers.

From a practical standpoint, the integrated framework offers decision-makers a structured, expert-informed tool for allocating resources more strategically. Rather than investing uniformly across all resilience domains, organizations may use these prioritization results to focus on high-leverage drivers that can generate systemic improvements across interconnected digital operations. The framework is particularly suited to contexts where quantitative data is scarce and decisions must rely on expert judgment under conditions of uncertainty. However, these findings should be interpreted with appropriate caution. As discussed in Section 3.6, the results are derived from expert perceptions and reflect perceived influence structures rather than empirically validated causal relationships. The prioritization outcomes are contingent upon the composition of the expert panel and the contextual boundaries of this study. Consequently, the proposed framework should be viewed as a decision-support instrument rather than a prescriptive or universally generalizable model.

Future research may extend this work in several directions. First, larger and more diverse expert panels spanning different industries, organizational sizes, and national contexts could enhance the generalizability of the findings. Second, longitudinal studies could examine how the relative importance of cyber-resilience drivers evolves over time in response to emerging threat landscapes and technological advancements. Third, integrating this framework with empirical organizational data and quantitative performance indicators could provide opportunities for validation and refinement. Comparative studies employing alternative multi-criteria decision-making methods may further enrich the understanding of cyber-resilience prioritization in AI-enabled digital ecosystems.

## Data Availability

The original contributions presented in the study are included in the article/Supplementary material, further inquiries can be directed to the corresponding author/s.
