# Supplementary material for: Cyber-resilience drivers in digital business networks: an expert-based prioritization using dual-gray DEMATEL and adaptive SWARA
Source: Front Artif Intell. 2026 Aug 5;9:1863919. doi: 10.3389/frai.2026.1863919 (PMC13486219; doi:10.3389/frai.2026.1863919)
Supplement: Supplementary file 1 [file Supplementary_file_1.docx]

**APPENDIX A. *Expert Evaluation Questionnaire***

Table A1. Expert profile form

| Item | Question | Response Options |
| --- | --- | --- |
| 1 | Current Position | □ Academic Researcher □ Cybersecurity Manager □ IT Manager □ Risk Management Specialist □ Security Consultant □ Other: ______ |
| 2 | Years of Professional Experience | □ 5–10 years □ 11–15 years □ 16–20 years □ More than 20 years |
| 3 | Area of Expertise | □ Cybersecurity □ Information Systems □ Digital Transformation □ Risk Management □ Organizational Resilience □ Other: ______ |
| 4 | Highest Academic Qualification | □ Bachelor’s Degree □ Master’s Degree □ PhD □ Other: ______ |
| 5 | Organization Type | □ Public Sector □ Private Sector □ Academic Institution □ Technology Company □ Financial Institution □ Other: ______ |

Table A2. Linguistic evaluation scale used for expert judgments

| Linguistic Evaluation | Symbol | Grey Interval |
| --- | --- | --- |
| No Influence | NI | [0,0] |
| Very Low Influence | VLI | [0,1] |
| Low Influence | LI | [1,2] |
| Moderate Influence | MI | [2,3] |
| High Influence | HI | [3,4] |
| Very High Influence | VHI | [4,5] |

Table A3. Definitions of cyber‑resilience drivers

| Code | Driver | Operational Description |
| --- | --- | --- |
| C1 | Cybersecurity Governance | Organizational capability to establish cybersecurity policies, oversight mechanisms, and strategic security management practices |
| C2 | Incident Response Capability | Ability to detect, respond to, and recover from cybersecurity incidents effectively |
| C3 | Risk Assessment and Monitoring | Continuous identification, evaluation, and monitoring of cyber risks and vulnerabilities |
| C4 | Employee Cybersecurity Awareness | Employees’ knowledge, training, and awareness regarding cybersecurity practices and threats |
| C5 | Business Continuity Planning | Organizational preparedness to maintain operations during and after cyber disruptions |
| C6 | Threat Intelligence Integration | Capability to collect, analyze, and integrate cyber threat intelligence into decision‑making processes |
| C7 | Data Protection and Privacy | Measures implemented to ensure confidentiality, integrity, and privacy of organizational data |
| C8 | Technological Infrastructure Flexibility | Adaptability and resilience of technological systems and digital infrastructure |
| C9 | Regulatory Compliance Capability | Ability to comply with cybersecurity laws, regulations, and industry standards |
| C10 | Information Sharing and Collaboration | Extent of collaboration and secure information exchange among ecosystem stakeholders |

Table A4. Pairwise influence assessment matrix used in the Dual‑Grey DEMATEL evaluation

Instruction:(Please determine the degree to which each driver influences another driver using the linguistic scale provided in Table A2.)

| **Drivers** | **C1** | **C2** | **C3** | **C4** | **C5** | **C6** | **C7** | **C8** | **C9** | **C10** |
| --- | --- | --- | --- | --- | --- | --- | --- | --- | --- | --- |
| C1 |  |  |  |  |  |  |  |  |  |  |
| C2 |  |  |  |  |  |  |  |  |  |  |
| C3 |  |  |  |  |  |  |  |  |  |  |
| C4 |  |  |  |  |  |  |  |  |  |  |
| C5 |  |  |  |  |  |  |  |  |  |  |
| C6 |  |  |  |  |  |  |  |  |  |  |
| C7 |  |  |  |  |  |  |  |  |  |  |
| C8 |  |  |  |  |  |  |  |  |  |  |
| C9 |  |  |  |  |  |  |  |  |  |  |
| C10 |  |  |  |  |  |  |  |  |  |  |

**APPENDIX B. *Computational Matrices***

Table B1. Aggregated grey direct-relation matrix (Lower bound $A^{L}$)

|  | C1 | C2 | C3 | C4 | C5 | C6 | C7 | C8 | C9 | C10 | C11 | C12 | C13 | C14 | C15 | C16 | C17 |
| --- | --- | --- | --- | --- | --- | --- | --- | --- | --- | --- | --- | --- | --- | --- | --- | --- | --- |
| C1 | 0 | 0.25 | 0.25 | 0.25 | 0.50 | 0.25 | 0.25 | 0.25 | 0.25 | 0.50 | 0.25 | 0.25 | 0.25 | 0.25 | 0.25 | 0.25 | 0.25 |
| C2 | 0.25 | 0 | 0.25 | 0.25 | 0.50 | 0.25 | 0.25 | 0.25 | 0.25 | 0.50 | 0.25 | 0.25 | 0.25 | 0.25 | 0.25 | 0.25 | 0.25 |
| C3 | 0.25 | 0.25 | 0 | 0.25 | 0.25 | 0.25 | 0.25 | 0.50 | 0.25 | 0.25 | 0.25 | 0.25 | 0.25 | 0.25 | 0.25 | 0.25 | 0.25 |
| C4 | 0.25 | 0.25 | 0.25 | 0 | 0.25 | 0.25 | 0.25 | 0.25 | 0.25 | 0.50 | 0.25 | 0.25 | 0.25 | 0.25 | 0.25 | 0.25 | 0.25 |
| C5 | 0.50 | 0.50 | 0.25 | 0.50 | 0 | 0.50 | 0.50 | 0.25 | 0.50 | 0.75 | 0.50 | 0.50 | 0.50 | 0.50 | 0.50 | 0.50 | 0.50 |
| C6 | 0.25 | 0.25 | 0.25 | 0.25 | 0.50 | 0 | 0.50 | 0.50 | 0.50 | 0.25 | 0.25 | 0.25 | 0.25 | 0.25 | 0.25 | 0.25 | 0.25 |
| C7 | 0.25 | 0.25 | 0.25 | 0.25 | 0.50 | 0.50 | 0 | 0.50 | 0.50 | 0.50 | 0.25 | 0.25 | 0.25 | 0.25 | 0.25 | 0.25 | 0.25 |
| C8 | 0.25 | 0.25 | 0.50 | 0.25 | 0.25 | 0.50 | 0.50 | 0 | 0.25 | 0.25 | 0.25 | 0.25 | 0.25 | 0.25 | 0.25 | 0.25 | 0.25 |
| C9 | 0.25 | 0.25 | 0.25 | 0.25 | 0.50 | 0.50 | 0.50 | 0.25 | 0 | 0.50 | 0.25 | 0.25 | 0.25 | 0.25 | 0.25 | 0.25 | 0.25 |
| C10 | 0.50 | 0.50 | 0.50 | 0.50 | 0.75 | 0.25 | 0.50 | 0.25 | 0.50 | 0 | 0.75 | 0.75 | 0.75 | 0.50 | 0.50 | 0.50 | 0.50 |
| C11 | 0.25 | 0.25 | 0.25 | 0.25 | 0.50 | 0.25 | 0.25 | 0.25 | 0.25 | 0.50 | 0 | 0.50 | 0.50 | 0.25 | 0.25 | 0.25 | 0.25 |
| C12 | 0.25 | 0.25 | 0.25 | 0.25 | 0.50 | 0.25 | 0.25 | 0.25 | 0.25 | 0.50 | 0.50 | 0 | 0.50 | 0.25 | 0.25 | 0.25 | 0.25 |
| C13 | 0.25 | 0.25 | 0.25 | 0.25 | 0.50 | 0.25 | 0.25 | 0.25 | 0.25 | 0.75 | 0.50 | 0.50 | 0 | 0.25 | 0.25 | 0.25 | 0.25 |
| C14 | 0.25 | 0.25 | 0.25 | 0.25 | 0.50 | 0.25 | 0.25 | 0.25 | 0.25 | 0.50 | 0.25 | 0.25 | 0.25 | 0 | 0.50 | 0.50 | 0.50 |
| C15 | 0.25 | 0.25 | 0.25 | 0.25 | 0.50 | 0.25 | 0.25 | 0.25 | 0.25 | 0.50 | 0.25 | 0.25 | 0.25 | 0.50 | 0 | 0.50 | 0.50 |
| C16 | 0.25 | 0.25 | 0.25 | 0.25 | 0.50 | 0.25 | 0.25 | 0.25 | 0.25 | 0.50 | 0.25 | 0.25 | 0.25 | 0.50 | 0.50 | 0 | 0.50 |
| C17 | 0.25 | 0.25 | 0.25 | 0.25 | 0.50 | 0.25 | 0.25 | 0.25 | 0.25 | 0.50 | 0.25 | 0.25 | 0.25 | 0.50 | 0.50 | 0.50 | 0 |

|  | C1 | C2 | C3 | C4 | C5 | C6 | C7 | C8 | C9 | C10 | C11 | C12 | C13 | C14 | C15 | C16 | C17 |
| --- | --- | --- | --- | --- | --- | --- | --- | --- | --- | --- | --- | --- | --- | --- | --- | --- | --- |
| C1 | 0 | 0.50 | 0.50 | 0.50 | 0.75 | 0.50 | 0.50 | 0.50 | 0.50 | 0.75 | 0.50 | 0.50 | 0.50 | 0.50 | 0.50 | 0.50 | 0.50 |
| C2 | 0.50 | 0 | 0.50 | 0.50 | 0.75 | 0.50 | 0.50 | 0.50 | 0.50 | 0.75 | 0.50 | 0.50 | 0.50 | 0.50 | 0.50 | 0.50 | 0.50 |
| C3 | 0.50 | 0.50 | 0 | 0.50 | 0.50 | 0.50 | 0.50 | 0.75 | 0.50 | 0.50 | 0.50 | 0.50 | 0.50 | 0.50 | 0.50 | 0.50 | 0.50 |
| C4 | 0.50 | 0.50 | 0.50 | 0 | 0.50 | 0.50 | 0.50 | 0.50 | 0.50 | 0.75 | 0.50 | 0.50 | 0.50 | 0.50 | 0.50 | 0.50 | 0.50 |
| C5 | 0.75 | 0.75 | 0.50 | 0.75 | 0 | 0.75 | 0.75 | 0.50 | 0.75 | 1.00 | 0.75 | 0.75 | 0.75 | 0.75 | 0.75 | 0.75 | 0.75 |
| C6 | 0.50 | 0.50 | 0.50 | 0.50 | 0.75 | 0 | 0.75 | 0.75 | 0.75 | 0.50 | 0.50 | 0.50 | 0.50 | 0.50 | 0.50 | 0.50 | 0.50 |
| C7 | 0.50 | 0.50 | 0.50 | 0.50 | 0.75 | 0.75 | 0 | 0.75 | 0.75 | 0.75 | 0.50 | 0.50 | 0.50 | 0.50 | 0.50 | 0.50 | 0.50 |
| C8 | 0.50 | 0.50 | 0.75 | 0.50 | 0.50 | 0.75 | 0.75 | 0 | 0.50 | 0.50 | 0.50 | 0.50 | 0.50 | 0.50 | 0.50 | 0.50 | 0.50 |
| C9 | 0.50 | 0.50 | 0.50 | 0.50 | 0.75 | 0.75 | 0.75 | 0.50 | 0 | 0.75 | 0.50 | 0.50 | 0.50 | 0.50 | 0.50 | 0.50 | 0.50 |
| C10 | 0.75 | 0.75 | 0.75 | 0.75 | 1.00 | 0.50 | 0.75 | 0.50 | 0.75 | 0 | 1.00 | 1.00 | 1.00 | 0.75 | 0.75 | 0.75 | 0.75 |
| C11 | 0.50 | 0.50 | 0.50 | 0.50 | 0.75 | 0.50 | 0.50 | 0.50 | 0.50 | 0.75 | 0 | 0.75 | 0.75 | 0.50 | 0.50 | 0.50 | 0.50 |
| C12 | 0.50 | 0.50 | 0.50 | 0.50 | 0.75 | 0.50 | 0.50 | 0.50 | 0.50 | 0.75 | 0.75 | 0 | 0.75 | 0.50 | 0.50 | 0.50 | 0.50 |
| C13 | 0.50 | 0.50 | 0.50 | 0.50 | 0.75 | 0.50 | 0.50 | 0.50 | 0.50 | 1.00 | 0.75 | 0.75 | 0 | 0.50 | 0.50 | 0.50 | 0.50 |
| C14 | 0.50 | 0.50 | 0.50 | 0.50 | 0.75 | 0.50 | 0.50 | 0.50 | 0.50 | 0.75 | 0.50 | 0.50 | 0.50 | 0 | 0.75 | 0.75 | 0.75 |
| C15 | 0.50 | 0.50 | 0.50 | 0.50 | 0.75 | 0.50 | 0.50 | 0.50 | 0.50 | 0.75 | 0.50 | 0.50 | 0.50 | 0.75 | 0 | 0.75 | 0.75 |
| C16 | 0.50 | 0.50 | 0.50 | 0.50 | 0.75 | 0.50 | 0.50 | 0.50 | 0.50 | 0.75 | 0.50 | 0.50 | 0.50 | 0.75 | 0.75 | 0 | 0.75 |
| C17 | 0.50 | 0.50 | 0.50 | 0.50 | 0.75 | 0.50 | 0.50 | 0.50 | 0.50 | 0.75 | 0.50 | 0.50 | 0.50 | 0.75 | 0.75 | 0.75 | 0 |

Table B2. Aggregated grey direct‑relation matrix (Upper bound $A^{U}$(

Table B3. Normalized lower matrix $D^{L}=s^{L}A^{L}$

|  | C1 | C2 | C3 | C4 | C5 | C6 | C7 | C8 | C9 | C10 | C11 | C12 | C13 | C14 | C15 | C16 | C17 |
| --- | --- | --- | --- | --- | --- | --- | --- | --- | --- | --- | --- | --- | --- | --- | --- | --- | --- |
| C1 | 0.000 | 0.029 | 0.029 | 0.029 | 0.059 | 0.029 | 0.029 | 0.029 | 0.029 | 0.059 | 0.029 | 0.029 | 0.029 | 0.029 | 0.029 | 0.029 | 0.029 |
| C2 | 0.029 | 0.000 | 0.029 | 0.029 | 0.059 | 0.029 | 0.029 | 0.029 | 0.029 | 0.059 | 0.029 | 0.029 | 0.029 | 0.029 | 0.029 | 0.029 | 0.029 |
| C3 | 0.029 | 0.029 | 0.000 | 0.029 | 0.029 | 0.029 | 0.029 | 0.059 | 0.029 | 0.029 | 0.029 | 0.029 | 0.029 | 0.029 | 0.029 | 0.029 | 0.029 |
| C4 | 0.029 | 0.029 | 0.029 | 0.000 | 0.029 | 0.029 | 0.029 | 0.029 | 0.029 | 0.059 | 0.029 | 0.029 | 0.029 | 0.029 | 0.029 | 0.029 | 0.029 |
| C5 | 0.059 | 0.059 | 0.029 | 0.059 | 0.000 | 0.059 | 0.059 | 0.029 | 0.059 | 0.088 | 0.059 | 0.059 | 0.059 | 0.059 | 0.059 | 0.059 | 0.059 |
| C6 | 0.029 | 0.029 | 0.029 | 0.029 | 0.059 | 0.000 | 0.059 | 0.059 | 0.059 | 0.029 | 0.029 | 0.029 | 0.029 | 0.029 | 0.029 | 0.029 | 0.029 |
| C7 | 0.029 | 0.029 | 0.029 | 0.029 | 0.059 | 0.059 | 0.000 | 0.059 | 0.059 | 0.059 | 0.029 | 0.029 | 0.029 | 0.029 | 0.029 | 0.029 | 0.029 |
| C8 | 0.029 | 0.029 | 0.059 | 0.029 | 0.029 | 0.059 | 0.059 | 0.000 | 0.029 | 0.029 | 0.029 | 0.029 | 0.029 | 0.029 | 0.029 | 0.029 | 0.029 |
| C9 | 0.029 | 0.029 | 0.029 | 0.029 | 0.059 | 0.059 | 0.059 | 0.029 | 0.000 | 0.059 | 0.029 | 0.029 | 0.029 | 0.029 | 0.029 | 0.029 | 0.029 |
| C10 | 0.059 | 0.059 | 0.059 | 0.059 | 0.088 | 0.029 | 0.059 | 0.029 | 0.059 | 0.000 | 0.088 | 0.088 | 0.088 | 0.059 | 0.059 | 0.059 | 0.059 |
| C11 | 0.029 | 0.029 | 0.029 | 0.029 | 0.059 | 0.029 | 0.029 | 0.029 | 0.029 | 0.059 | 0.000 | 0.059 | 0.059 | 0.029 | 0.029 | 0.029 | 0.029 |
| C12 | 0.029 | 0.029 | 0.029 | 0.029 | 0.059 | 0.029 | 0.029 | 0.029 | 0.029 | 0.059 | 0.059 | 0.000 | 0.059 | 0.029 | 0.029 | 0.029 | 0.029 |
| C13 | 0.029 | 0.029 | 0.029 | 0.029 | 0.059 | 0.029 | 0.029 | 0.029 | 0.029 | 0.088 | 0.059 | 0.059 | 0.000 | 0.029 | 0.029 | 0.029 | 0.029 |
| C14 | 0.029 | 0.029 | 0.029 | 0.029 | 0.059 | 0.029 | 0.029 | 0.029 | 0.029 | 0.059 | 0.029 | 0.029 | 0.029 | 0.000 | 0.059 | 0.059 | 0.059 |
| C15 | 0.029 | 0.029 | 0.029 | 0.029 | 0.059 | 0.029 | 0.029 | 0.029 | 0.029 | 0.059 | 0.029 | 0.029 | 0.029 | 0.059 | 0.000 | 0.059 | 0.059 |
| C16 | 0.029 | 0.029 | 0.029 | 0.029 | 0.059 | 0.029 | 0.029 | 0.029 | 0.029 | 0.059 | 0.029 | 0.029 | 0.029 | 0.059 | 0.059 | 0.000 | 0.059 |
| C17 | 0.029 | 0.029 | 0.029 | 0.029 | 0.059 | 0.029 | 0.029 | 0.029 | 0.029 | 0.059 | 0.029 | 0.029 | 0.029 | 0.059 | 0.059 | 0.059 | 0.000 |

Table B4. Normalized upper matrix $D^{U}=s^{U}A^{U}$

|  | C1 | C2 | C3 | C4 | C5 | C6 | C7 | C8 | C9 | C10 | C11 | C12 | C13 | C14 | C15 | C16 | C17 |
| --- | --- | --- | --- | --- | --- | --- | --- | --- | --- | --- | --- | --- | --- | --- | --- | --- | --- |
| C1 | 0.000 | 0.040 | 0.040 | 0.040 | 0.060 | 0.040 | 0.040 | 0.040 | 0.040 | 0.060 | 0.040 | 0.040 | 0.040 | 0.040 | 0.040 | 0.040 | 0.040 |
| C2 | 0.040 | 0.000 | 0.040 | 0.040 | 0.060 | 0.040 | 0.040 | 0.040 | 0.040 | 0.060 | 0.040 | 0.040 | 0.040 | 0.040 | 0.040 | 0.040 | 0.040 |
| C3 | 0.040 | 0.040 | 0.000 | 0.040 | 0.040 | 0.040 | 0.040 | 0.060 | 0.040 | 0.040 | 0.040 | 0.040 | 0.040 | 0.040 | 0.040 | 0.040 | 0.040 |
| C4 | 0.040 | 0.040 | 0.040 | 0.000 | 0.040 | 0.040 | 0.040 | 0.040 | 0.040 | 0.060 | 0.040 | 0.040 | 0.040 | 0.040 | 0.040 | 0.040 | 0.040 |
| C5 | 0.060 | 0.060 | 0.040 | 0.060 | 0.000 | 0.060 | 0.060 | 0.040 | 0.060 | 0.080 | 0.060 | 0.060 | 0.060 | 0.060 | 0.060 | 0.060 | 0.060 |
| C6 | 0.040 | 0.040 | 0.040 | 0.040 | 0.060 | 0.000 | 0.060 | 0.060 | 0.060 | 0.040 | 0.040 | 0.040 | 0.040 | 0.040 | 0.040 | 0.040 | 0.040 |
| C7 | 0.040 | 0.040 | 0.040 | 0.040 | 0.060 | 0.060 | 0.000 | 0.060 | 0.060 | 0.060 | 0.040 | 0.040 | 0.040 | 0.040 | 0.040 | 0.040 | 0.040 |
| C8 | 0.040 | 0.040 | 0.060 | 0.040 | 0.040 | 0.060 | 0.060 | 0.000 | 0.040 | 0.040 | 0.040 | 0.040 | 0.040 | 0.040 | 0.040 | 0.040 | 0.040 |
| C9 | 0.040 | 0.040 | 0.040 | 0.040 | 0.060 | 0.060 | 0.060 | 0.040 | 0.000 | 0.060 | 0.040 | 0.040 | 0.040 | 0.040 | 0.040 | 0.040 | 0.040 |
| C10 | 0.060 | 0.060 | 0.060 | 0.060 | 0.080 | 0.040 | 0.060 | 0.040 | 0.060 | 0.000 | 0.080 | 0.080 | 0.080 | 0.060 | 0.060 | 0.060 | 0.060 |
| C11 | 0.040 | 0.040 | 0.040 | 0.040 | 0.060 | 0.040 | 0.040 | 0.040 | 0.040 | 0.060 | 0.000 | 0.060 | 0.060 | 0.040 | 0.040 | 0.040 | 0.040 |
| C12 | 0.040 | 0.040 | 0.040 | 0.040 | 0.060 | 0.040 | 0.040 | 0.040 | 0.040 | 0.060 | 0.060 | 0.000 | 0.060 | 0.040 | 0.040 | 0.040 | 0.040 |
| C13 | 0.040 | 0.040 | 0.040 | 0.040 | 0.060 | 0.040 | 0.040 | 0.040 | 0.040 | 0.080 | 0.060 | 0.060 | 0.000 | 0.040 | 0.040 | 0.040 | 0.040 |
| C14 | 0.040 | 0.040 | 0.040 | 0.040 | 0.060 | 0.040 | 0.040 | 0.040 | 0.040 | 0.060 | 0.040 | 0.040 | 0.040 | 0.000 | 0.060 | 0.060 | 0.060 |
| C15 | 0.040 | 0.040 | 0.040 | 0.040 | 0.060 | 0.040 | 0.040 | 0.040 | 0.040 | 0.060 | 0.040 | 0.040 | 0.040 | 0.060 | 0.000 | 0.060 | 0.060 |
| C16 | 0.040 | 0.040 | 0.040 | 0.040 | 0.060 | 0.040 | 0.040 | 0.040 | 0.040 | 0.060 | 0.040 | 0.040 | 0.040 | 0.060 | 0.060 | 0.000 | 0.060 |
| C17 | 0.040 | 0.040 | 0.040 | 0.040 | 0.060 | 0.040 | 0.040 | 0.040 | 0.040 | 0.060 | 0.040 | 0.040 | 0.040 | 0.060 | 0.060 | 0.060 | 0.000 |

Table B5. Total-relation matrix (lower bound) $T^{L}$

|  | C1 | C2 | C3 | C4 | C5 | C6 | C7 | C8 | C9 | C10 | C11 | C12 | C13 | C14 | C15 | C16 | C17 |
| --- | --- | --- | --- | --- | --- | --- | --- | --- | --- | --- | --- | --- | --- | --- | --- | --- | --- |
| C1 | 0.087 | 0.098 | 0.094 | 0.093 | 0.135 | 0.097 | 0.102 | 0.097 | 0.102 | 0.141 | 0.102 | 0.102 | 0.104 | 0.102 | 0.104 | 0.104 | 0.104 |
| C2 | 0.098 | 0.087 | 0.094 | 0.093 | 0.135 | 0.097 | 0.102 | 0.097 | 0.102 | 0.141 | 0.102 | 0.102 | 0.104 | 0.102 | 0.104 | 0.104 | 0.104 |
| C3 | 0.094 | 0.094 | 0.079 | 0.089 | 0.122 | 0.095 | 0.100 | 0.110 | 0.100 | 0.127 | 0.100 | 0.100 | 0.101 | 0.100 | 0.101 | 0.101 | 0.101 |
| C4 | 0.093 | 0.093 | 0.089 | 0.079 | 0.122 | 0.095 | 0.100 | 0.095 | 0.100 | 0.133 | 0.100 | 0.100 | 0.101 | 0.100 | 0.101 | 0.101 | 0.101 |
| C5 | 0.130 | 0.130 | 0.117 | 0.128 | 0.105 | 0.128 | 0.133 | 0.117 | 0.133 | 0.173 | 0.133 | 0.133 | 0.135 | 0.133 | 0.135 | 0.135 | 0.135 |
| C6 | 0.100 | 0.100 | 0.095 | 0.095 | 0.135 | 0.082 | 0.116 | 0.116 | 0.116 | 0.126 | 0.103 | 0.103 | 0.104 | 0.103 | 0.104 | 0.104 | 0.104 |
| C7 | 0.102 | 0.102 | 0.097 | 0.097 | 0.138 | 0.116 | 0.085 | 0.119 | 0.119 | 0.145 | 0.106 | 0.106 | 0.108 | 0.106 | 0.108 | 0.108 | 0.108 |
| C8 | 0.098 | 0.098 | 0.110 | 0.095 | 0.125 | 0.113 | 0.118 | 0.082 | 0.105 | 0.131 | 0.103 | 0.103 | 0.104 | 0.103 | 0.104 | 0.104 | 0.104 |
| C9 | 0.102 | 0.102 | 0.097 | 0.097 | 0.138 | 0.119 | 0.124 | 0.105 | 0.085 | 0.145 | 0.106 | 0.106 | 0.108 | 0.106 | 0.108 | 0.108 | 0.108 |
| C10 | 0.141 | 0.141 | 0.135 | 0.135 | 0.178 | 0.128 | 0.145 | 0.122 | 0.145 | 0.141 | 0.158 | 0.158 | 0.160 | 0.145 | 0.147 | 0.147 | 0.147 |
| C11 | 0.102 | 0.102 | 0.097 | 0.097 | 0.138 | 0.103 | 0.106 | 0.103 | 0.106 | 0.145 | 0.085 | 0.119 | 0.121 | 0.106 | 0.108 | 0.108 | 0.108 |
| C12 | 0.102 | 0.102 | 0.097 | 0.097 | 0.138 | 0.103 | 0.106 | 0.103 | 0.106 | 0.145 | 0.119 | 0.085 | 0.121 | 0.106 | 0.108 | 0.108 | 0.108 |
| C13 | 0.104 | 0.104 | 0.101 | 0.101 | 0.141 | 0.104 | 0.108 | 0.104 | 0.108 | 0.160 | 0.121 | 0.121 | 0.087 | 0.108 | 0.110 | 0.110 | 0.110 |
| C14 | 0.102 | 0.102 | 0.097 | 0.097 | 0.138 | 0.103 | 0.106 | 0.103 | 0.106 | 0.145 | 0.106 | 0.106 | 0.108 | 0.082 | 0.116 | 0.116 | 0.116 |
| C15 | 0.104 | 0.104 | 0.101 | 0.101 | 0.141 | 0.104 | 0.108 | 0.104 | 0.108 | 0.147 | 0.108 | 0.108 | 0.110 | 0.116 | 0.087 | 0.119 | 0.119 |
| C16 | 0.104 | 0.104 | 0.101 | 0.101 | 0.141 | 0.104 | 0.108 | 0.104 | 0.108 | 0.147 | 0.108 | 0.108 | 0.110 | 0.116 | 0.119 | 0.087 | 0.119 |
| C17 | 0.104 | 0.104 | 0.101 | 0.101 | 0.141 | 0.104 | 0.108 | 0.104 | 0.108 | 0.147 | 0.108 | 0.108 | 0.110 | 0.116 | 0.119 | 0.119 | 0.087 |

Table B6. Total-relation matrix (Upper bound) $T^{U}$

|  | C1 | C2 | C3 | C4 | C5 | C6 | C7 | C8 | C9 | C10 | C11 | C12 | C13 | C14 | C15 | C16 | C17 |
| --- | --- | --- | --- | --- | --- | --- | --- | --- | --- | --- | --- | --- | --- | --- | --- | --- | --- |
| C1 | 0.119 | 0.136 | 0.130 | 0.129 | 0.167 | 0.135 | 0.141 | 0.135 | 0.141 | 0.178 | 0.141 | 0.141 | 0.144 | 0.141 | 0.144 | 0.144 | 0.144 |
| C2 | 0.136 | 0.119 | 0.130 | 0.129 | 0.167 | 0.135 | 0.141 | 0.135 | 0.141 | 0.178 | 0.141 | 0.141 | 0.144 | 0.141 | 0.144 | 0.144 | 0.144 |
| C3 | 0.130 | 0.130 | 0.106 | 0.123 | 0.150 | 0.132 | 0.138 | 0.154 | 0.138 | 0.159 | 0.138 | 0.138 | 0.141 | 0.138 | 0.141 | 0.141 | 0.141 |
| C4 | 0.129 | 0.129 | 0.123 | 0.106 | 0.150 | 0.132 | 0.138 | 0.132 | 0.138 | 0.168 | 0.138 | 0.138 | 0.141 | 0.138 | 0.141 | 0.141 | 0.141 |
| C5 | 0.165 | 0.165 | 0.149 | 0.163 | 0.135 | 0.163 | 0.170 | 0.149 | 0.170 | 0.208 | 0.170 | 0.170 | 0.173 | 0.170 | 0.173 | 0.173 | 0.173 |
| C6 | 0.138 | 0.138 | 0.132 | 0.132 | 0.167 | 0.111 | 0.158 | 0.158 | 0.158 | 0.160 | 0.145 | 0.145 | 0.147 | 0.145 | 0.147 | 0.147 | 0.147 |
| C7 | 0.141 | 0.141 | 0.135 | 0.135 | 0.170 | 0.158 | 0.114 | 0.162 | 0.162 | 0.185 | 0.148 | 0.148 | 0.151 | 0.148 | 0.151 | 0.151 | 0.151 |
| C8 | 0.135 | 0.135 | 0.154 | 0.132 | 0.153 | 0.155 | 0.162 | 0.111 | 0.145 | 0.167 | 0.145 | 0.145 | 0.147 | 0.145 | 0.147 | 0.147 | 0.147 |
| C9 | 0.141 | 0.141 | 0.135 | 0.135 | 0.170 | 0.162 | 0.170 | 0.145 | 0.114 | 0.185 | 0.148 | 0.148 | 0.151 | 0.148 | 0.151 | 0.151 | 0.151 |
| C10 | 0.178 | 0.178 | 0.172 | 0.172 | 0.212 | 0.160 | 0.185 | 0.154 | 0.185 | 0.184 | 0.196 | 0.196 | 0.200 | 0.185 | 0.188 | 0.188 | 0.188 |
| C11 | 0.141 | 0.141 | 0.135 | 0.135 | 0.170 | 0.145 | 0.148 | 0.145 | 0.148 | 0.185 | 0.114 | 0.162 | 0.165 | 0.148 | 0.151 | 0.151 | 0.151 |
| C12 | 0.141 | 0.141 | 0.135 | 0.135 | 0.170 | 0.145 | 0.148 | 0.145 | 0.148 | 0.185 | 0.162 | 0.114 | 0.165 | 0.148 | 0.151 | 0.151 | 0.151 |
| C13 | 0.144 | 0.144 | 0.141 | 0.141 | 0.173 | 0.147 | 0.151 | 0.147 | 0.151 | 0.200 | 0.165 | 0.165 | 0.119 | 0.151 | 0.154 | 0.154 | 0.154 |
| C14 | 0.141 | 0.141 | 0.135 | 0.135 | 0.170 | 0.145 | 0.148 | 0.145 | 0.148 | 0.185 | 0.148 | 0.148 | 0.151 | 0.111 | 0.158 | 0.158 | 0.158 |
| C15 | 0.144 | 0.144 | 0.141 | 0.141 | 0.173 | 0.147 | 0.151 | 0.147 | 0.151 | 0.188 | 0.151 | 0.151 | 0.154 | 0.158 | 0.119 | 0.162 | 0.162 |
| C16 | 0.144 | 0.144 | 0.141 | 0.141 | 0.173 | 0.147 | 0.151 | 0.147 | 0.151 | 0.188 | 0.151 | 0.151 | 0.154 | 0.158 | 0.162 | 0.119 | 0.162 |
| C17 | 0.144 | 0.144 | 0.141 | 0.141 | 0.173 | 0.147 | 0.151 | 0.147 | 0.151 | 0.188 | 0.151 | 0.151 | 0.154 | 0.158 | 0.162 | 0.162 | 0.119 |
